# The quality, accuracy and appropriateness of UK optometric age‐related macular degeneration referrals

**DOI:** 10.1111/opo.13455

**Published:** 2025-02-07

**Authors:** Corinne Fulcher, Christopher Davey, Jonathan Denniss

**Affiliations:** ^1^ Centre for Vision across the Life Span University of Huddersfield Huddersfield UK; ^2^ Bradford Ophthalmic Research Network Bradford Teaching Hospitals NHS Foundation Trust Bradford UK; ^3^ School of Optometry and Vision Science University of Bradford Bradford UK

**Keywords:** accuracy, AMD, macular, optometrist, quality, referral

## Abstract

**Purpose:**

Little is known about the quality of optometrists' referrals to secondary care for neovascular age‐related macular degeneration (nAMD), despite the need for timely intervention. We analysed the content and accuracy of optometrists' referrals for nAMD. Adherence to UK National Institute for Health and Care Excellence (NICE) guidelines and the impact of the COVID‐19 pandemic were assessed as secondary measures.

**Methods:**

Optometric referrals to a specialist macular treatment centre in Bradford, United Kingdom, between March 2019 and March 2021 were retrospectively analysed and compared with subsequent electronic medical records. Data were extracted on legibility, reason for referral, patient and optometrist demographics, visual acuity, reported signs and symptoms, patient diagnosis and patient outcomes. Binomial logistic regression models were constructed to determine whether signs or symptoms noted in the referral were associated with subsequent nAMD diagnosis in secondary care and whether optometrist gender or experience influenced nAMD referral accuracy.

**Results:**

Across all 394 referrals analysed, 256 were for nAMD. Referral accuracy for nAMD was 39.8% (95% CI [34.0%, 45.9%]), with the most common reason for misdiagnosis being dry AMD. However, 76.8% of patients referred for suspected nAMD were either treated in secondary care or observed over multiple visits. 20% of suspected nAMD patients were seen within the NICE recommended 14‐day window pre‐COVID, dropping to 5% during the pandemic (*p* < 0.001). Visual acuity was most strongly associated with nAMD diagnosis (χ^2^(1) = 13.71, *p* < 0.001) followed by macular haemorrhage (χ^2^(1) = 5.89, *p* = 0.02). Neither optometrist gender nor experience was significantly associated with confirmed nAMD. Legibility of referrals was 91–95% for patient details and 94–97% for the referring optometrist.

**Conclusions:**

Although the overall quality and legibility of optometrists' macular referrals to secondary care were of a high standard, the diagnostic accuracy of nAMD was below 40%. Referred visual acuity was the main sign/symptom associated with confirmed nAMD diagnosis.

**Key points**

- Optometric referral accuracy of neovascular age‐related macular degeneration was below 40%, but 77% of referrals were deemed necessary based on patient outcome.
- Legibility and content of optometric macular referrals is of a high standard, with legibility consistently above 90%.
- Referred visual acuity is the main predictor of a confirmed neovascular age‐related macular degeneration diagnosis, followed by macular haemorrhage.

## INTRODUCTION

In the United Kingdom, an estimated 2.5% of people aged over 65 years have ‘wet’ or neovascular age‐related macular degeneration (nAMD).^1^ Treatment of nAMD with anti‐vascular endothelial growth factor (anti‐VEGF) drugs enables many patients to maintain or even improve vision from first presentation.^2^, ^3^, ^4^ However, the treatment is most effective when started early,^5^ hence clinical guidelines, such as those provided by the National Institute for Health and Care Excellence (NICE) in the United Kingdom, currently state that patients diagnosed with nAMD should be treated within 14 days of referral.^6^

In the United Kingdom, most patients noticing a change in vision present to an optometrist or general medical practitioner, who will then refer to secondary care if required. Referrals are typically triaged by secondary care centres, and therefore, the urgency with which a referred patient is seen is often dictated by the content of the referral letter. Around 2008, the Thames Valley Macular Group developed a ‘Wet AMD rapid access referral form’ (also known as the ‘nAMD fast track form’) which was endorsed by the Royal College of Ophthalmologists, Macular Society and Royal National Institute of Blind People to help speed up referrals for suspected nAMD. The use of this form has been widely adopted by primary care optometrists across the United Kingdom, and it has been incorporated into electronic referral platforms, yet literature describing how appropriately the form is being used remains scarce.

Although a number of studies have looked at overall quality and accuracy of optometry referrals into Ophthalmology departments,^7^, ^8^, ^9^ macular referrals typically make up a small proportion of these and subanalysis has not been provided. Data on diagnostic accuracy and adherence to NICE guidelines are likely to vary substantially depending on the reason for referral, and legibility data from studies over a decade ago^9^, ^10^ are less relevant now due to advances in electronic record keeping and referrals. Therefore, presently, little is known about the quality and accuracy of macular‐specific referrals from primary care optometrists to secondary care. This is in contrast with other ocular diseases such as glaucoma.^11^, ^12^, ^13^, ^14^, ^15^ Muen and Hewick^16^ reported on the accuracy and quality of optometry nAMD referrals to the Highlands National Health Service (NHS) trust using a rapid access referral form, but only a relatively small sample of 54 referrals were analysed. A correct diagnosis of nAMD was found in 37% of cases. Although optometrists were good at establishing symptoms of reduced vision and distortion, compared to an ophthalmologist, they were only correct in reporting drusen and subretinal fluid in around half of referred cases. Recently, a study in Wales reported only a 26% accuracy rate for optometric nAMD referrals, which improved to 56% when patients were triaged in a community referral refinement scheme.^17^

In March 2020, the World Health Organisation declared the novel coronavirus (COVID‐19) outbreak to be a global pandemic. In response, on the 26th March 2020, the United Kingdom implemented a nationwide lockdown, which saw businesses close and people asked to remain in their homes. With people unable or unwilling to visit an optometrist following the onset of new visual symptoms, it is likely that new cases of nAMD were not identified as quickly during the pandemic and referrals may have been delayed. Although optometric practices remained open during the pandemic for emergency and essential care, some utilised remote consultations or omitted certain tests which could also have affected referral content and accuracy.

Here, data are presented from optometric referrals into the Bradford Macular Centre (BMC), a specialist referral centre within an NHS hospital trust (Bradford Teaching Hospitals NHS Foundation Trust, Bradford, UK), set up to receive referrals for suspected macular disease requiring medical, rather than surgical intervention. The primary aim of the project was to audit the referrals for quality, accuracy and appropriateness, offering suggestions for improvement where possible. However, as this time frame also encompasses the start of the COVID‐19 pandemic and subsequent UK ‘lockdowns’, we also looked at the impact of the pandemic and the adherence to NICE guidelines^6^ as secondary measures.

## METHODS

A list of all new patients referred into the BMC between 1 March 2019 and 28 February 2021 was obtained from the BMC administrative team, and referrals originating from a primary care optometrist were extracted for further analysis. To study the possible impact of the COVID‐19 pandemic on both optometric referrals and referral outcomes, the group was then further subdivided into a ‘pre‐COVID’ group (referrals between 01 March 2019 and 28 February 2020) and a ‘COVID’ group (referrals between 01 March 2020 and 28 February 2021).

As this study was considered a retrospective service evaluation and data were collected as part of normal service delivery (before being fully anonymised), informed patient consent was not required. However, a member of the NHS clinical care team did check the hospital database to see whether patients had previously given consent for their medical records to be viewed, and only those who had given consent were included in the study. Local ethical approval for the study was approved by the University of Huddersfield's Research Integrity and Ethics Committee.

Referrals were excluded if (a) they were not an optometric referral, (b) the patient was never seen face to face at BMC, (c) no referral letter was attached to the electronic patient record, (d) the patient did not consent for their electronic medical records to be viewed by the care team, (e) the patient was not seen face to face for over 6 months from the referral being received (as the pathological status of the eye may have significantly changed), (f) the referral was not for a suspected macular condition and (g) the patient was already actively receiving treatment at BMC.

A member of the clinical care team at BMC (CF) retrieved relevant information from electronic copies of each referral letter. This included patient demographics, reason for referral, the study eye, patient symptoms (as reported by the optometrist), visual acuity (VA), suspected diagnosis, type of referral letter (nAMD fast‐track form or other), whether optical coherence tomography (OCT) was used/mentioned, optometrist name, practice and their professional registration number. Where a diagnosis was not explicitly mentioned but a nAMD fast track form was used, the implied reason for referral was recorded as nAMD. The study eye was taken as the referred eye. If both eyes were referred, or the referred eye was not explicitly stated, the eye with the worst visual acuity was taken (unless both visual acuities were the same, in which case the right eye was taken). Reported ‘vision loss’ was taken as any objective indication by the optometrist that the patient's vision had declined (e.g., explicitly stated or a comparison of previous and current acuity showing a reduction). ‘Blurred vision’ was then taken as any indication that the patient subjectively reported a reduction in vision (e.g., written description or by ticking the ‘blurred vision’ box). The legibility of patient and optometrist details was recorded (‘yes’ or ‘no’), and any missing data were automatically recorded as illegible. Legibility was determined as the ability to identify the patient's name and either address or date of birth, and optometrist's name or General Optical Council (GOC) registration number (such that they could be identified on the public register). The patient's final diagnosis and visual acuity following full assessment by a clinician at BMC was then obtained from their medical records for comparison. All data were fully anonymised on site prior to analysis by the research team.

As there are numerous ways to report visual acuity in UK clinical practice, and we were collecting data retrospectively, all acuity measurements were converted to LogMAR for comparison. This allowed non‐standard measurements (e.g., Snellen 6/8), Early Treatment of Diabetic Retinopathy letter scores, low vision scores (hand movements, count fingers, perception of light) and visual acuities with ±values (e.g., 6/9^−2^) to be standardised for statistical analysis. An assumption was made that each letter was equivalent to a LogMAR value of 0.02. We recognise that this may have introduced a small error into our acuity values and the real score per letter could have fallen in the range ±0.0125 to 0.05 (assuming 2–8 letters per row). However, it is impossible to know how many letters were presented at the time visual acuity was measured, and the error should be relatively small. Low vision scores were converted to LogMAR using the reference values published by the National Ophthalmology Database.^18^

Days to first appointment was determined as the number of full calendar days between the referral being received by the BMC and the patient being seen in a face‐to‐face clinic. When determining whether there were any significant differences in duration during the COVID pandemic, any patients who had cancelled appointments themselves or failed to attend were omitted from the analysis. This was to show the impact of COVID on hospital‐centred rather than patient‐centred appointment delays. To evaluate adherence to NICE guidelines,^6^ we looked at how many patients received their first appointment within the recommended 14‐day window. This again excluded any patients who chose to cancel or rearrange their first offered appointment or failed to attend. At the BMC, patients requiring anti‐VEGF treatment are offered an injection on the same day (one‐stop service), and thus, this ‘time to appointment’ metric is useful in determining how many patients could have received treatment within the recommended 14 days.

Optometrist gender and registration with the General Optical Council was then retrieved from the public register (https://str.optical.org/). The number of years registered with the GOC was determined as the difference in years between the date of registration on the register and 31 March 2022 (an arbitrary date chosen during data analysis to provide consistency).

Two separate binomial logistic regression models were constructed for the pre‐COVID and COVID data sets with the aim of assessing which clinical features of the referrals were associated with nAMD diagnosis. Models were constructed and analysed with the open‐source statistical software *R* (version 4.2.1, r‐project.org). For this analysis, only patients referred for nAMD were considered, and three patients (two pre‐COVID, one COVID) were excluded who did not have visual acuity recorded. The following predictors were added to the model by forward stepwise addition in this order: visual acuity, vision loss reported (yes/no), blurred vision reported (yes/no), distortion reported (yes/no), fluid reported (yes/no), haemorrhage reported (yes/no), exudate reported (yes/no), drusen reported (yes/no). Models were compared to the null or previous model by the ꭓ^2^ likelihood ratio test, with predictors being accepted for inclusion when model fit improved with *p* < 0.05. Nagelkerke's *R* ^2^ was used to estimate overall model fit.^19^ Further separate models were constructed in the same way to assess the association of optometrist years since registration and gender with nAMD referral accuracy in the pre‐COVID and COVID cohorts. For these analyses, two COVID referrals were excluded since the optometrist's details were not legible.

All other statistical analysis was conducted using IBM SPSS Statistics (Version 28, ibm.com/spss), and statistical significance was assumed at *p* < 0.05. Mean values were compared using *t*‐tests, and categorical data were compared using ꭓ^2^ tests. 95% confidence intervals for proportions were calculated using the Wilson score interval to account for small sample sizes in certain categories.

## RESULTS

Initially, 489 referrals were obtained from the BMC. Of these, 95 were excluded because they were not an optometric referral (*n* = 12), were never seen at BMC (*n* = 38), no referral letter was available (*n* = 20), patient did not consent for their medical records to be viewed (*n* = 9), patient not seen in person for over 6 months (and hence pathological status of eye could have significantly changed) (*n* = 11), not a macular referral (*n* = 2) or an existing patient already undergoing treatment (*n* = 3). This left 394 referrals for retrospective analysis, which could be subdivided into pre‐COVID (*n* = 208) and COVID (*n* = 186) groups.

### Patient demographics

Patient demographic data are presented in Table 1 for both study groups, alongside information about the referring optometrist/optometric practice. One referral in the COVID group did not state the referring practice and was excluded from ‘referring practice’ analysis. Comparing the demographics of the pre‐COVID and COVID groups, there were no significant differences in mean age, ethnicity (as clinically recorded), % female gender or type of referring practice as determined by Pearson's χ^2^ test or *t*‐test.

**TABLE 1:** Comparing the patient and optometrist demographics and referring practices between the Pre‐COVID and COVID groups. nAMD, neovascular age‐related macular degeneration.

	Pre‐COVID group (*n* = 208)	COVID group (*n* = 186)	*p*‐Value (χ^2^ or *t*‐test)
Ethnicity			0.30
White	166 (79.8%)	158 (84.9%)	
Asian	23 (11.1%)	18 (9.5%)	
Other	19 (9.1%)	10 (5.4%)	
Mean age	73.7 years (SD 14.3 years)	72.6 years (SD 13.8 years)	0.47
Patient gender			0.81
Female	116 (55.8%)	106 (57.0%)	
Male	92 (44.2%)	80 (43.0%)	
Referring practice type			0.21
Multiple	130 (62.5%)	123 (66.1%)	
Independent	62 (29.8%)	56 (29.6%)	
Domiciliary	16 (7.7%)	6 (3.2%)	
Unknown		1 (0.5%)	
Reason for referral			0.55
Suspected nAMD	138 (66.3%)	118 (63.4%)	
Other	70 (33.7%)	68 (36.6%)	
Referring optometrist gender			0.37
Female	129 (62.0%)	107 (57.5%)	
Male	76 (36.5%)	76 (40.9%)	
Unknown	3 (1.4%)	3 (1.6%)	
Referring optometrist years registered (median)	12 years (range: 1–41 years)	10 years (range: <1–43 years)	0.15

Abbreviation: AMD, neovascular age‐related macular degeneration.

### Appropriateness

The reasons for referral were categorised as nAMD (*n* = 256), retinal vein occlusion (*n* = 29), macular oedema (*n* = 16), central serous retinopathy (*n* = 12), non‐specific maculopathy (*n* = 63), vitreoretinal/structural cases (*n* = 14) and other (*n* = 4). Fast‐track forms were used in 254 (64.5%) of all referrals to the BMC, including 25 (6.3%) which were non‐nAMD referrals. The non‐nAMD referrals sent on a fast‐track form were categorised as branch retinal vein occlusion (*n* = 7), cystoid macular oedema (*n* = 1), diabetic macular oedema (*n* = 1), central serous retinopathy (*n* = 9) and macular hole/vitreomacular traction (*n* = 7), as explicitly stated on the referral letter. 27 (6.8%) nAMD referrals were made without use of the fast‐track form, although this did include six who did not meet the NICE eligibility criteria for treatment.^6^

### Legibility and content

Across all 394 referrals, the overall legibility of the referral letters was high across all four measures: 95.4% for patient name and date of birth (95% CI [93.0%, 97.5]), 90.6% for patient address (95% CI [87.3%, 93.1%]), 96.7% for optometrist name (95% CI [94.4%, 98.1%]) and 93.7% for optometrist practice (95% CI [90.8%, 95.7%]), with no significant differences noted between the pre‐COVID and COVID groups as determined by Pearson's ꭓ^2^ test (patient name and date of birth (*p* = 0.81), patient address (*p* = 0.06), optometrist name (*p* = 0.11) and practice address (*p* = 0.74)). A further 25 referrals (11 pre‐COVID and 14 COVID) included a partial address (e.g., street name only), which if included increased overall address legibility to 96.7% (95% CI [94.4%, 98.1%]). For the pre‐COVID and COVID groups, optometrists included distance visual acuity measures in the affected eye in 206 (99.0%) (95% CI [96.6%, 99.7%]) and 182 (97.8%) referrals (95% CI [94.6%, 99.2%]), respectively, and near visual acuity measures in the affected eye in 159 (76.4%) (95% CI [70.2%, 81.7%]) and 134 (72.0%) referrals (95% CI [65.2%, 78.0%]), respectively. Pearson's χ^2^ found no significant difference between the reporting of visual acuity between the groups (distance VA, *p* = 0.34; near VA, *p* = 0.32) suggesting similar reporting during the pandemic. However, OCT data were reported in 19 (9.1%) (95% CI [5.9%, 13.8%]) and 44 (23.7%) referrals (95% CI [18.1%, 30.3%]) for pre‐COVID and COVID, respectively, which was a significant increase (*p* < 0.001).

The nAMD fast‐track form requires the referring clinician to indicate whether the patient is experiencing a number of signs and/or symptoms which may be linked to nAMD. Table 2 shows the frequency with which each sign/symptom was positively reported. Patients who were subsequently diagnosed with nAMD at their secondary care appointment (‘confirmed nAMD’) are shown separately (combined across pre‐COVID and COVID groups).

**TABLE 2:** Frequency of reported symptoms on the referrals.

Symptoms	Entire cohort	Pre‐COVID (*n* = 208)	COVID (*n* = 186)	All confirmed nAMD patients (*n* = 118)
Vision loss	274 (69.5%)	142 (68.2%)	132 (71.0%)	97 (82.2%)
Blurred vision	207 (52.5%)	108 (51.9%)	99 (53.2%)	68 (57.6%)
Distortion	199 (50.5%)	103 (49.5%)	96 (51.6%)	64 (54.2%)
Haemorrhage	146 (37.1%)	90 (43.3%)	56 (30.1%)	57 (48.3%)
Drusen	135 (34.3%)	82 (39.4%)	53 (28.5%)	55 (46.6%)
Macular fluid	177 (44.9%)	77 (37.0%)	100 (53.8%)	61 (51.7%)
Exudate	46 (11.7%)	22 (10.6%)	24 (12.9%)	17 (14.4%)

Abbreviation: nAMD, neovascular age‐related macular degeneration.

To determine whether these reported signs/symptoms, in conjunction with other data from the referral, were associated with the likelihood of the patient having nAMD, a binomial logistic regression model was used. This analysis considered only referrals for nAMD. In the pre‐COVID data, there were 135 referrals of whom 54 (40%) were confirmed as nAMD. In the COVID data, there were 117 referrals, of whom 47 (40%) were confirmed as nAMD. For pre‐COVID data, both visual acuity (χ^2^(1) = 13.71, *p* < 0.001) and haemorrhage (χ^2^(1) = 5.89, *p* = 0.02) significantly improved the model, whereas the other tested signs/symptoms did not. Details of the final model are shown in Table 3, and the full stepwise addition results are provided in the Data S1. In the same analysis for the COVID data, only visual acuity significantly improved the model (χ^2^(1) = 21.66, *p* < 0.001).

**TABLE 3:** The binomial logistic regression model testing associations between nAMD diagnosis and referral content for pre‐COVID and COVID data.

	*B* (SE)	*p*‐Value	Odds ratio (95% CI)
**Pre‐COVID (*n* = 135, 54 [40%] confirmed nAMD)**			
Constant	−1.62 (0.37)		
Visual acuity (per 1.00 logMAR increase)	1.35 (0.47)	0.004	3.85 (1.66–10.47)
Haemorrhage present	0.91 (0.38)	0.02	2.49 (1.19–5.29)
*R* ^2^ = 0.18 (Nagelkerke), Model χ^2^(2) = 19.60, *p* < 0.001			
**COVID (*n* = 117, 47 [40%] confirmed nAMD)**			
Constant	−1.48 (0.33)		
Visual acuity (per 1.00 logMAR increase)	1.55 (0.39)	<0.001	4.71 (2.33–10.91)
*R* ^2^ = 0.23 (Nagelkerke), Model χ^2^(1) = 21.66, *p* < 0.001			

Abbreviation: nAMD, neovascular age‐related macular degeneration.

### Accuracy

**FIGURE 1:**
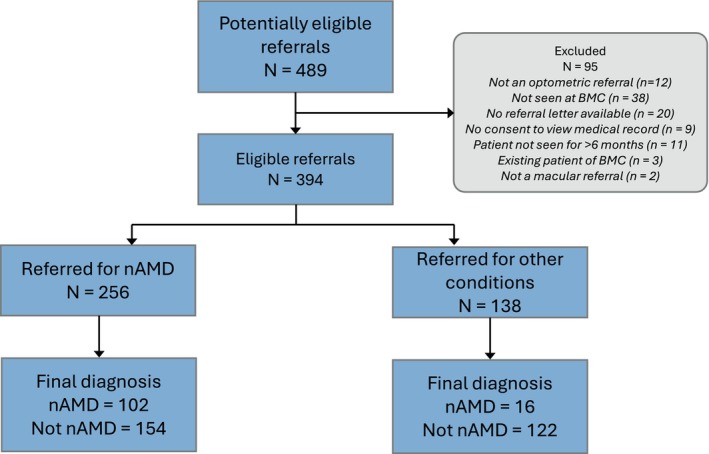
A summary flow diagram of referrals and final outcome. BMC, Bradford Macular Centre; nAMD, neovascular age‐related macular degeneration.

The accuracy of optometric referrals was determined where a specific diagnosis was stated (or implied through the use of a nAMD fast‐track form *n* = 191/256), by comparing the optometrist's suggested diagnosis against the final diagnosis from secondary care. Since sample sizes of non‐nAMD referrals were generally small (ranging from 4 to 29), data from the pre‐COVID and COVID groups were combined. Accuracy was defined as the proportion of referrals for a given condition subsequently confirmed as having that condition. Accuracy was highest for central serous retinopathy at 91.7% (95% CI [64.6%, 98.5%]) and lowest for nAMD at 39.8% (95% CI [34.0%, 45.9%]). As nAMD was of particular interest in this study, Figure 1 shows a summary flowchart highlighting the true and false positives for nAMD referral accuracy. The subsequent proportion of false‐positive referrals for nAMD (i.e., proportion of nAMD referrals that were *not* confirmed as nAMD) was 60.1%.

The accuracy of other referred conditions was 79.3% for retinal vein occlusion (95% CI [61.6%, 90.2%]), 43.8% for macular oedema (95% CI [23.1%, 66.8%]) and 85.7% for combined vitreomacular surgical disorders, including macular hole, macular traction and epiretinal membrane (95% CI [60.1%, 96.0%]).

**FIGURE 2:**
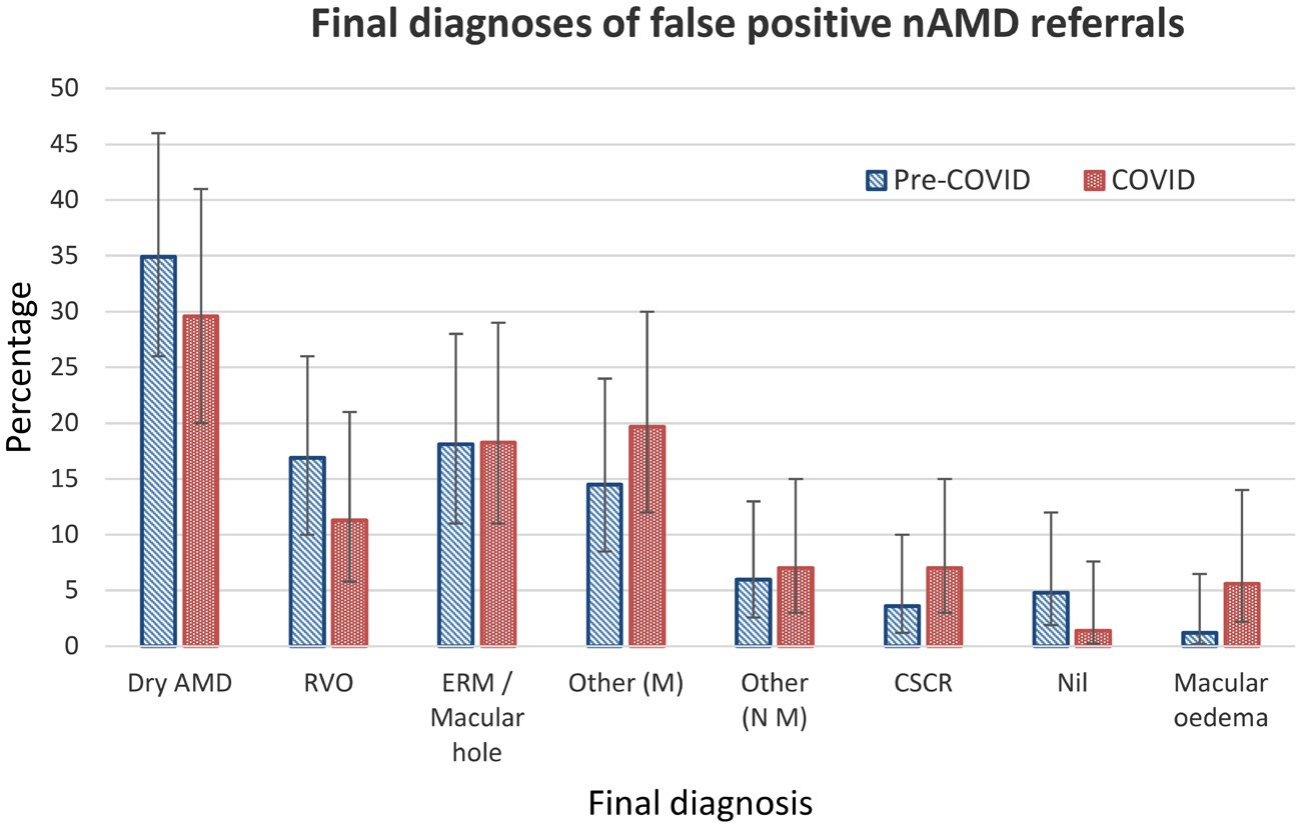
Final diagnosis of all 154 false‐positive nAMD referrals (AMD, age‐related macular degeration; CSCR, central serous chorioretinopathy; ERM, epiretinal membrane; nAMD, neovascular age‐related macular degeneration; Nil, no pathology detected; Other (M), other maculopathy; Other (NM), other non‐maculopathy; RVO, retinal vein occlusion). Error bars show the 95% confidence intervals. Frequencies for each condition are shown in Table S1.

As the sample size for the nAMD referrals was considerably larger (*n* = 256) and this gave the poorest referral accuracy, subanalysis was performed to find the most common source of error (see Figure 2). The most common reason for misdiagnosis of nAMD was found to be dry AMD, which may not be surprising given that this is often a precursor to neovascular changes and can have similar fundus characteristics. A breakdown of the frequency of each ‘misdiagnosis’ is given in Table S1.

### Referring optometrist information

Referrals were made by a male optometrist in 37.3% of pre‐COVID cases and 41.5% of cases during the COVID pandemic. Although it appears that fewer referrals were sent by male than female optometrists, this may simply reflect the lower percentage of male registrants in the United Kingdom, currently standing at a ratio of ~2:3 males to females.^20^ Comparing how long the referring optometrists had been registered with the General Optical Council, the median time was 12 years (range: 1–41 years) in the pre‐COVID group and 10 years (range: <1 to 43 years) in the COVID group. In the binomial logistic regression, neither gender (*p* = 0.96 pre‐COVID, *p* = 0.53 COVID) nor years registered (*p* = 0.05 pre‐COVID, *p* = 0.49 COVID) were significantly associated with a confirmed nAMD diagnosis. Whether the optometrist specified a tentative diagnosis on the referral was also examined, as this may have been more difficult during the pandemic due to the aforementioned restrictions and change in clinical practice. For this analysis we did not assume that anything sent on the fast‐track form was a nAMD referral, and only included the referral in our analysis if a diagnosis was mentioned explicitly. The percentage of optometrists mentioning a specific diagnosis increased significantly from 21.6% pre‐COVID to 36.6% during the pandemic (Pearson's χ^2^ test, *p* = 0.002).

### Referral outcomes

For patients referred for possible nAMD (‘suspected nAMD’), the mean duration between the referral and the first appointment in secondary care was 28.4 days (SD 15.1 days) pre‐pandemic. During COVID, this increased significantly to 37.8 days (SD 20.1 days) (*p* < 0.001). Only 20.2% of suspected nAMD patients were seen within 14 days before the pandemic, and this decreased significantly to 5.1% during the pandemic (*p* = 0.001).

**FIGURE 3:**
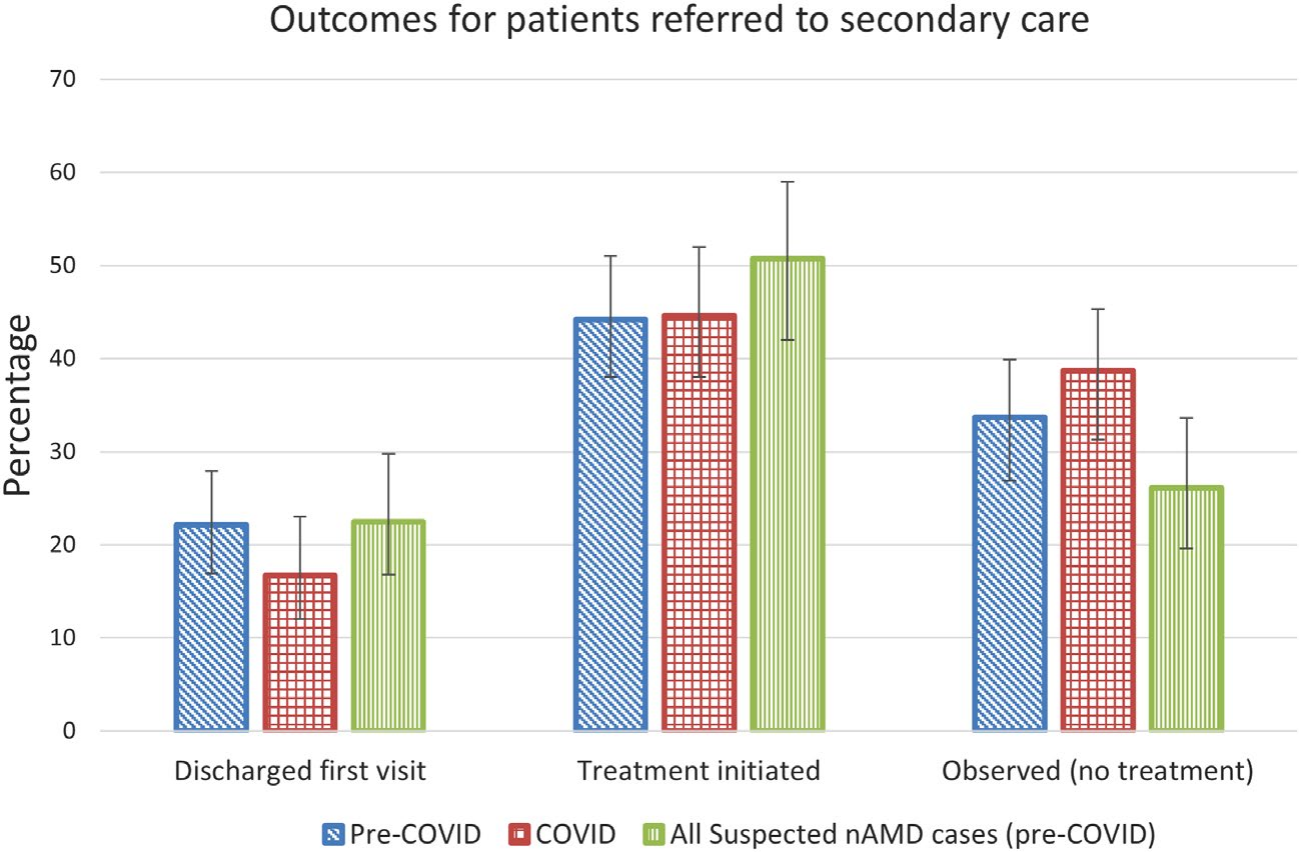
Patient outcomes for all 394 patients (pre‐COVID and COVID) referred to the Bradford Macular Centre (BMC). Patients were either discharged at their first visit with no treatment (*n* = 77/394), treated (*n* = 175/394) or observed over more than one visit without ever receiving treatment (*n* = 142/394). The outcomes of all suspected nAMD cases (pre‐COVID only) are also shown (*n* = 138). Error bars show 95% confidence intervals. The specific frequencies of each outcome are given in Table S2. nAMD, neovascular age‐related macular degeneration.

Once seen in secondary care, Figure 3 shows the proportion of all referred patients who were discharged at the first visit without treatment, compared with the proportion who were either treated or observed over several visits (without treatment) by the secondary care team. Of the entire cohort, it was noted that five patients had vision that was too poor to be eligible for treatment, and nine were offered treatment and declined. Before COVID, around one in five patients was discharged without treatment at their first visit, and this number reduced to one in six during the pandemic, with more being monitored before discharge. A breakdown of the exact frequencies is given in Table S2. The number receiving treatment remained stable across both groups at ~44%. Pearson's χ^2^ showed no significant difference between the secondary care outcomes before and during the pandemic (*p* = 0.38).

As the accuracy of nAMD diagnosis was relatively poor amongst optometry referrals, the patients who were referred for possible nAMD (‘suspected nAMD’) were examined further (see Figure 3). Only the pre‐COVID group was selected as these patients were receiving the usual standard of care and were not impacted by pandemic‐related changes in procedure. Just over a quarter of these patients were observed across more than one visit without ever receiving treatment, perhaps indicating that the diagnosis of nAMD can still be uncertain even within a secondary care setting. Of the treated patients, it is also worth noting that four were listed for cataract surgery or YAG capsulotomy, one was treated for macular oedema and one was listed for an epiretinal membrane peel, and hence, they were not diagnosed with (and treated for) nAMD.

## DISCUSSION

The vast majority of referrals sent to the BMC were for suspected nAMD, followed by ‘non‐specific maculopathy’ where the optometrist did not specify a potential diagnosis but reported signs and symptoms of macular pathology and did not use the fast‐track form. The nAMD fast‐track referral form was used for 89.5% of the suspected nAMD cases and 6.8% of non‐nAMD cases.

If one simply compares the referred eye condition with the diagnosis from the hospital, the overall accuracy of nAMD referrals was similar to the 37% accuracy reported by Muen and Hewick,^16^ and slightly higher than the recently reported 26% accuracy by Sanders et al.^17^ This would suggest that the ability of primary care optometrists to recognise and diagnose nAMD has not improved significantly in the last decade. It is also far lower than the *overall* optometric referral accuracy rates reported as ranging between 76%^21^ and 98%,^8^ although both studies only included a small sample of nAMD/retina referrals. Pierscionek et al. also reported 75% accuracy in optometric diagnosis (and referral) of ‘retinal/vitreous’ disease, although they did not define exactly which conditions this encompassed.^22^ That being said, ~27% of suspected nAMD cases in the present study required multiple secondary care visits (pre‐pandemic) before being discharged without treatment, so even within the ophthalmology department, nAMD may be a difficult condition to diagnose correctly. Although optometric *diagnostic* accuracy may be relatively low at ~39%, many nAMD referrals are still warranted, as most of these referred patients do require the additional diagnostic testing, clinical expertise and, in some cases, treatment afforded by secondary care. When examining only the suspected nAMD referrals from before the pandemic, and including all cases where the patient was either treated or observed over multiple visits, results from the present study suggest 76.8% of nAMD referrals were *necessary*.

In addition to calculating the false‐positive rate for nAMD referral accuracy (60.1%, see Figure 1), the number of ‘discharged first visit’ patients may also give an indication of ‘false positives’ for the entire cohort (394 referrals). This metric is different because it assumes a referral was only warranted if they were treated or observed in secondary care, and was calculated as 19.3% (across all referred conditions). However, it should be remembered that nine patients declined treatment and five did not meet treatment criteria, some of whom were discharged immediately and some were observed for a while. Also, the cohort included *all* patients referred to secondary care, and not all of the referred conditions would require ophthalmic treatment (e.g., mild diabetic macular oedema).

Neither optometrist gender nor experience (years registered) were significantly associated with nAMD referral accuracy, potentially contradicting previous studies which found newly qualified and female optometrists were more likely to generate false‐positive referrals.^7^, ^23^ One possible reason is that the present study focused on macular referrals, and macular pathology such as nAMD is known to be associated with significant sight loss which increases with time to treatment.^24^ As such, it is possible that more optometrists, irrespective of gender or experience, may err on the side of caution and refer, compared with other conditions such as suspect glaucoma or cataract where experienced clinicians may be happier to monitor patients in practice.

The binomial logistic regression model found that poorer visual acuity increased the likelihood of a correct nAMD diagnosis, with the presence of haemorrhage also increasing the likelihood in the pre‐COVID group. This may have been because fundus examinations were somewhat limited at the start of the pandemic, and hence haemorrhages may have been missed in the COVID group. It could be argued that every other metric on the referral form is superfluous to nAMD referral accuracy, although they may be clinically useful in other ways. For example, metamorphopsia has previously been shown to correlate well with ‘treatment‐requiring AMD’.^25^ Additionally, as some clinical findings can be transient, having a record of what was seen by the optometrist at the patient's first presentation can be a useful part of their history.

One factor affecting the quality and accuracy of referrals may be a lack of financial motivation, as some optometrists may feel they are not being adequately compensated for providing additional diagnostic tests or monitoring signs and symptoms in practice.^26^ It has also been suggested that inadequate feedback from secondary care providers may have a significant impact on referral quality.^8^, ^27^, ^28^ Despite guidelines from the Royal College of Ophthalmologists stating that feedback on nAMD referrals must be sent to the referrer,^29^ in a recent study where nAMD made up 7.5% of all optometric referrals, the referral reply rate was only 2.6%^28^ Without meaningful feedback, community optometrists do not have the opportunity to learn whether their proposed diagnosis was accurate and if the referral was necessary. Feedback from retinal specialists in secondary care should also be helpful when developing new skills such as OCT interpretation. There is a scarcity of interventional studies looking at how improving the feedback loop could impact referral quality, possibly due to known barriers within secondary care such as dated technology systems, financial motivations to report to the patient's general medical practitioner instead of their optometrist and time pressures in busy clinics.^28^ Yet this evidence may be necessary to help implement change to policies, funding, communication channels or otherwise, and as such is suggested to be an important focus going forwards. The implementation of referral refinement schemes may also help to ease the pressure on secondary care, as a recent study in Wales showed that the positive referral rate for nAMD more than doubled when patients were triaged by a trained community optometrist before being seen at the hospital.^17^ However, it is vital that such services are appropriately funded and supported by secondary care.

Future research should consider ways to improve nAMD diagnostic accuracy among community optometrists. A recent study by Parkins et al.^30^ found that continued education and training (CET) had no significant effect on referral practice, although they did indicate that their sample was subject to selection bias as participants had undertaken significantly more CET and had completed more peer discussions than a reference sample. It could be valuable to analyse whether the increasing availability of OCT data is improving diagnostic accuracy among optometrists. A vignette study by Jindal et al.^31^ found OCT data significantly improved diagnostic performance among optometrists, compared with fundus photographs alone. Yet this may not necessarily translate to real‐world judgements as their participants did receive training beforehand and made all clinical judgements during a single hour‐long session where they knew that they were assessing images for signs of nAMD. Grace et al. also recently developed and tested an online tool for improving the diagnosis of central macular lesions, reporting improved recognition and interpretation, particularly among optometrists who identified as ‘novice’ OCT users.^32^ However, it is important to note that while the introduction of more OCT machines into primary care may improve diagnostic accuracy (and reduce false‐positive referrals), it *could* also increase the number of referrals through increased incidental findings.

The referring optometrist could be identified in all but nine (2.3%) of the 394 referrals assessed in the present study, from either their registration number, name or both. This appears to be an improvement over previous studies reporting 33%,^23^ 31%^33^ and 17.7%^10^ of optometric referrals containing an illegible or absent referrer name, although it should be noted that these studies did not state whether the GOC registration number could have been used to identify the optometrist in the absence of a name or practitioner code. It should also be noted that, in a very small number of cases (*n* = 5), the legibility of the patient and/or optometrist data was restricted by poor scanning of data or printing from the hospital fax machine, and not the fault of the referring clinician. As it is likely that increasing numbers of referrals are being sent electronically, this should become less of a problem in the future.

Across the entire cohort, 25 (6.3%) referrals only included the first line of the patient's address but still contained enough information to identify the patient reliably. Visual acuity for the affected eye was also reported in 99.0% of all pre‐COVID referrals, which is comparable to the study by Davey et al.,^10^ who reported the inclusion of visual acuity measures in 96% of optometrist referrals. This continued to be the case during the pandemic even when optometrists were operating under the Red and Amber phases of the College of Optometrists COVID‐19 Guidance for Professional Practice,^34^ with visual acuity being reported in 97.8% of referrals. During this time, optometrists were recommended to ‘adapt their routine to reduce close contact with patients and streamline consultations to only do tests that are clinically necessary’. This could potentially explain why OCT was mentioned on significantly more referrals during COVID, with optometrists choosing methods of investigation that did not require close proximity. Alternatively, it may indicate increased use of the technology, greater availability of OCT devices in optometric practice or simply greater reporting of OCT results.

There were some limitations to the present study including the aforementioned conversion of all acuity measurements to a LogMAR score. It was presumed that each letter was equivalent to a LogMAR score of 0.02. However, the real score per letter could have fallen in the range of ±0.0125 to 0.05 (assuming 2–8 letters per row). Additionally, the assumption was made that non‐specific referrals sent on a fast‐track form were for nAMD, when this may not have been the optometrist's intention. In a similar manner, any non‐specific referrals *not* sent on a fast‐track form were recorded as ‘non‐specific maculopathy’, when the optometrist may have intended these to be an nAMD referral. Both of these situations could have led to errors in the estimation of the proportion of true and false positives. Additionally, ‘vision loss’ was recorded as any objective indication by the optometrist that the patient's vision had declined (e.g., explicitly stated or a comparison of previous and current visual acuity showing a reduction), and ‘blurred vision’ as any indication that the patient subjectively reported a reduction in vision (e.g., written description or by ticking the ‘blurred vision’ box). However, it is uncertain if this is what the optometrist truly intended. The legibility results were consistently high, although it was not recorded whether the referrals were handwritten by the optometrist or typed, which obviously would have implications for legibility. Finally, since this study took place in a single geographic location, it is uncertain whether similar levels of accuracy would be obtained in other parts of the United Kingdom, or whether referral practices have changed in the years since data collection took place. Future research should aim to look at how generalisable the results are to different regions and try and include a greater number of non‐nAMD macular referrals to gain a better understanding of the accuracy of conditions such as central serous chorioretinopathy and macular oedema.

To our knowledge, this study is the first published report analysing the quality and content of optometric macular referrals in England, including 394 referrals in the analysis. Arguably nAMD is one of the most time‐critical ocular conditions, and hence, accurate and rapid referral from community to secondary care is crucial. The overall quality and legibility of optometric macular referrals in this study was of a high standard, although the diagnostic accuracy of nAMD has not shown improvement in the last decade. Understanding what potential barriers exist for primary care optometrists (e.g., time, technology, motivation) will be an important next step when looking to improve this figure. Nevertheless, around three quarters of optometric referrals were deemed necessary, as evidenced by the appointment outcome. The logistic regression model used here showed that reduced visual acuity was the main sign/symptom at referral associated with a positive nAMD diagnosis, and hence, primary care optometrists should pay particular attention to this when deciding whether nAMD is present. OCT‐based triaging clinics run by trained optometrists are now being utilised in secondary care, but whether OCT improves diagnostic accuracy of nAMD in primary care settings (negating the need for a referral) would be an interesting focus for future research.

## AUTHOR CONTRIBUTIONS

**Corinne Fulcher:** Conceptualization (lead); data curation (lead); formal analysis (equal); methodology (equal); project administration (lead); writing – original draft (lead); writing – review and editing (lead). **Christopher Davey:** Formal analysis (supporting); methodology (equal); writing – original draft (supporting); writing – review and editing (supporting). **Jonathan Denniss:** Conceptualization (supporting); formal analysis (equal); methodology (equal); writing – original draft (supporting); writing – review and editing (supporting).

## FUNDING INFORMATION

There was no funding attached to this study.

## CONFLICT OF INTEREST STATEMENT

The authors declare no conflict of interest.

## Supporting information


Data S1.

Table S1.
