# Supplementary material for: The quality, accuracy and appropriateness of UK optometric age‐related macular degeneration referrals
Source: Ophthalmic Physiol Opt. 2025 Feb 7;45(3):799–809. doi: 10.1111/opo.13455 (PMC11976509; doi:10.1111/opo.13455)
Supplement: Supplementary file 1 — Data S1. [file OPO-45-799-s001.docx]

**Supplementary Model Information**

The following represent the chi square likelihood ratio test results at each stage of the forward stepwise addition process used to build the binomial logistic regression models for association of referral features with confirmed diagnosis of nAMD (see methods section). Briefly, variables were added to the model sequentially, with those where p<0.05 (marked with * in the tables below) kept in the model for subsequent additions.

Pre-COVID data model:

| **Variable added to model** | **χ^2^(1)** | **p** |
| --- | --- | --- |
| Referred eye VA (logMAR) | 13.71 | <0.001* |
| Vision loss reported (yes/no) | 1.09 | 0.30 |
| Blurred vision reported (yes/no) | 1.64 | 0.20 |
| Distortion reported (yes/no) | 1.12 | 0.29 |
| Fluid reported (yes/no) | 3.01 | 0.08 |
| Haemorrhage reported (yes/no) | 5.89 | 0.015* |
| Exudate reported (yes/no) | 0.61 | 0.43 |
| Drusen reported (yes/no) | 0.007 | 0.93 |

COVID data model:

| **Variable added to model** | **χ^2^(1)** | **p** |
| --- | --- | --- |
| Referred eye VA (logMAR) | 21.66 | <0.001* |
| Vision loss reported (yes/no) | 2.68 | 0.10 |
| Blurred vision reported (yes/no) | 0.01 | 0.94 |
| Distortion reported (yes/no) | 1.54 | 0.21 |
| Fluid reported (yes/no) | 0.004 | 0.95 |
| Haemorrhage reported (yes/no) | 2.65 | 0.10 |
| Exudate reported (yes/no) | 0.64 | 0.42 |
| Drusen reported (yes/no) | 2.52 | 0.11 |
