# Supplementary material for: The quality, accuracy and appropriateness of UK optometric age‐related macular degeneration referrals
Source: Ophthalmic Physiol Opt. 2025 Feb 7;45(3):799–809. doi: 10.1111/opo.13455 (PMC11976509; doi:10.1111/opo.13455)
Supplement: Supplementary file 2 — Table S1. [file OPO-45-799-s002.docx]

**Supplementary Table 1:** A summary table for all 154 false positive nAMD referrals, showing the frequencies of the true diagnosis as documented in secondary care.

| **Final diagnosis** | **Pre-COVID frequency** | **COVID frequency** | **Total frequency** |
| --- | --- | --- | --- |
| Dry AMD | 29 (34.9%) | 21(29.6%) | 50 (32.5%) |
| Retinal vein occlusion | 14 (16.9%) | 8 (11.3%) | 22 (14.3%) |
| Epiretinal membrane / macular hole | 15 (18.1%) | 13 (18.3%) | 28 (18.2%) |
| Other (maculopathy) | 12 (14.5%) | 14 (19.7%) | 26 (16.9%) |
| Other (non-maculopathy) | 5 (6.0%) | 5 (7.0%) | 10 (6.5%) |
| Central serous chorioretinopathy | 3 (3.6%) | 5 (7.0%) | 8 (5.2%) |
| No pathology detected | 4 (4.8%) | 1 (1.4%) | 5 (3.2%) |
| Macular oedema | 1 (1.2%) | 4 (5.6%) | 5 (3.2%) |

**Supplementary Table 2:** A summary table of patient outcomes, showing the number who were discharged at the first visit, observed over 1 or more visits but never treated, and those who were treated.

| **Referral outcome** | **Pre-COVID frequency**  **(n = 208)** | **COVID frequency**  **(n = 186)** | **Total frequency**  **(n = 394)** | **Only suspected nAMD pre-COVID (n=138)** |
| --- | --- | --- | --- | --- |
| Discharged first visit | 46 (22.1%) | 31(16.7%) | 77 (19.5%) | 30 (21.7%) |
| Observed but never treated | 70 (33.7%) | 72 (38.7%) | 142 (36.0%) | 38 (27.5%) |
| Treated | 92 (44.2%) | 83 (44.6%) | 175 (44.4%) | 70 (50.7%) |
